# Hysterosalpingographic Appearances of Female Genital Tract Tuberculosis: Part I. Fallopian Tube

**Published:** 2013-12-22

**Authors:** Firoozeh Ahmadi, Fatemeh Zafarani, Gholam Shahrzad

**Keywords:** Female Genital Tuberculosis, Hysterosalpingography, Fallopian Tube

## Abstract

Female genital tuberculosis (TB) remains as a major cause of tubal obstruction leading to infertility, especially in developing countries. The global prevalence of genital tuberculosis has increased during the past two decades due to increasing acquired immunodeficiency syndrome (AIDS). Genital TB is commonly asymptomatic, and it is diagnosed during infertility investigations. Despite of recent advances in imaging tools, such as computerized tomography (CT) scan, magnetic resonance imaging (MRI) and ultrasongraphy, hysterosalpingography is still the standard screening test for evaluation of tubal infertility and a valuable tool for diagnosis of female genital tuberculosis. Tuberculosis gives rise to various appearances on hysterosalpingography (HSG) from non-specific changes to specific findings. The present pictorial review illustrates and describes specific and non-specific radiographic features of female genital tuberculosis in two parts. Part I presents specific findings of tuberculosis related to tubes such as "beaded tube", "golf club tube", "pipestem tube", "cobble stone tube" and "leopard skin tube". Part II describes adverse effects of tuberculosis on structure of endometrium and radiological specific findings such as "dwarfed" uterus with lymphatic intravasation and occluded tubes, "T-shaped" tuberculosis uterus, "pseudounicornuate" uterus and "Collar-stud abscess", which have not been encountered in the majority of non-tuberculosis cases.

## Introduction

Female genital tuberculosis (FGTB) is one form of extrapulmonary manifestations of tuberculosis, while it includes 5% of all female pelvic infections and 10% of pulmonary tuberculosis cases (1, 2). It is more frequent in developing countries, leading to chronic pelvic inflammatory disease (PID) and infertility (3).

The reported prevalence of genital tuberculosis has shown a descending trend in developed countries, but recently, its rate has started to increase again due to co-infection with human immunodeficiency virus (HIV) and the development of drug-resistant strains of Mycobacterium tuberculosis (4-6).

Primary infection of the female genital organs is very rare (7), and is usually secondary to infection of elsewhere in the body, usually the lungs (8, 9).

Diagnosis of genital TB may be difficult because majority of cases are asymptomatic; in addition, facilities for mycobacterium culture and histopathology are limited in high-prevalence countries (9-11). In these circumstances, the infection is usually diagnosed during hysterosalpingography for preliminary investigations of infertility (12, 13). In addition, hysterosalpingography is still the golden standard for evaluation of tubal lumen (14) and a helpful procedure in diagnosis of female genital tuberculosis (15, 16). Genital tuberculosis gives rise to various appearances on hysterosalpingography (HSG) from non-specific changes to specific findings.

This pictorial review describes specific and nonspecific radiographic features of tubes caused by tuberculosis as seen on HSG.

### Pathology of fallopian tube tuberculosis

Mycobacterium tuberculosis is responsible for disease in approximately 90-95% of cases and produces granulomatous salpingitis and endometritis leading irregular menstrual bleeding and infertility. In 5-10% of patients, the infection results from Mycobacterium bovis, especially when the source of infection is acquired from the gastrointestinal cases (17, 18).

Genital tuberculosis usually spread to genital site from three routes, including hematogenous, lymphatic or adjacent viscera (19), while it most commonly affects the fallopian tubes (95-100%), followed by the endometrium (50-60%), ovaries (20-30%), cervix (5-15%), and vulva/vagina (1%) and the myometrium (2.5%) (20, 21).

Primary infection of genital TB is rare, and may result from direct introduction of TB bacilli at sexual intercourse with a male partner with genitourinary TB. Ascending spread of infection from the vagina, cervix and the vulva has been reported (22).

Tha fallopian tubes are the initial focus of female genital tuberculosis, and usually involved bilaterally not symmetrically (23). The pathological changes related to tube vary according to severity of disease. The histological hallmark of TB salpingitis is the epitheliod cell granuloma.

The transitional region between the isthmus and ampulla is the most frequent site of tubal obstruction. Sometimes, hydrosalpinx or pyosalpinx with thick fibrotic wall is formed at the distally blocked fallopian tube.The ovaries are often seen in normal appearance and the diagnosis is established only on histopathological studies (23).

In some cases, ovarian tubercle, adhesion, capsular thickening and ovarian or pelvic abscess are formed (24).

### Clinical presentation

Most of the cases involved in genital TB have been detected in reproductive age; a range of 20- 45 year-old (25). Genital TB may be presented with a spectrum of clinical symptoms, but the disease is usually asymptomatic, and it is preliminary diagnosed during infertility investigations (11, 12). The most common clinical symptom are infertility, pelvic mass and abnormal uterine bleeding (26-28). In the acute phase, PID with pelvic pain, fever and vaginal discharge may be seen.

Tuberculous lesions of the cervix present with postcoital bleeding, abnormal discharge and, on examination, have appearances similar to cancer of the cervix (11). Involvement of the ovaries may result in an adnexal ovarian mass. Fistula formation to the bowel, skin or vagina may be seen. Peritoneal involvement may give rise to ascites.

### Hysterosalpingographic finding of tubal tuberculosis

Hysterosalpingographic presentation of tubal TB vary from non-specific changes such as hydrosalpinx to specific pattern such as "beaded tube", "golf club tube", "pipestem tube", "cobble stone tube" and the "leopard skin tube" (14). Of course some clinicians have assumed that these features may be non-specific for tuberculosis but are highly suggestive of (16, 29). There are useful differential diagnostic criteria suggested by Klein et al. (29) for diagnosis of tuberculosis.

### Calcifications

**Fig 1:**
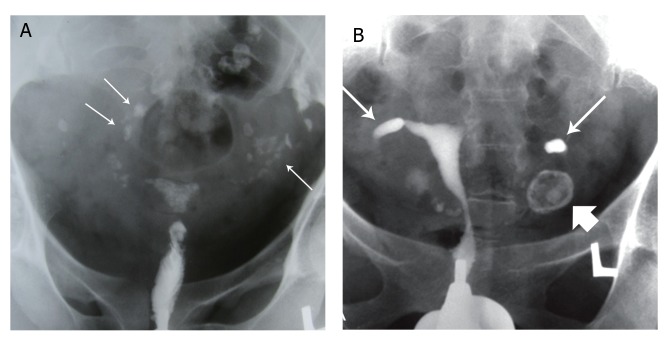
A. Multiple calcified lymph nodes in a patient with chronic genital tuberculosis. Calcified lymph nodes are present in the pelvic (long thin arrows) and course of fallopian tubes. Note the irregularity of the uterine contour and diminished uterine capacity, B. Hysterogram shows a large shadow of a calcified ovary (open arrow) in the left side. Hydrosalpinx is also seen in the tubal distal portion in the both tubes (32).

The presence calcified lymph nodes in the pelvis or in the course of fallopian tubes may enable the diagnosis of the TB. Plain films of the pelvis may show such calcifications which must be differentiated from other causes of calcifications such as calcified pelvic nodes, calcified uterine myomas, urinary calculi, pelvic phleboliths and calcification in an ovarian dermoid (8). The calcified lymph node presents as a single or multiple round, irregular or mulberry feature (Fig 1A, B). Tubal calcification are usually seen in the form of small linear streaks laying in the course of tubes (30). Sometimes, they may be straight, bent, or curved in shape. Tuberculous tubo-ovarian abscesses may present in one or both sides, and sometimes are seen as well-defined masses. Occasionally denser areas are seen due to granulomas (31).

### Tubal outline

**Fig 2:**
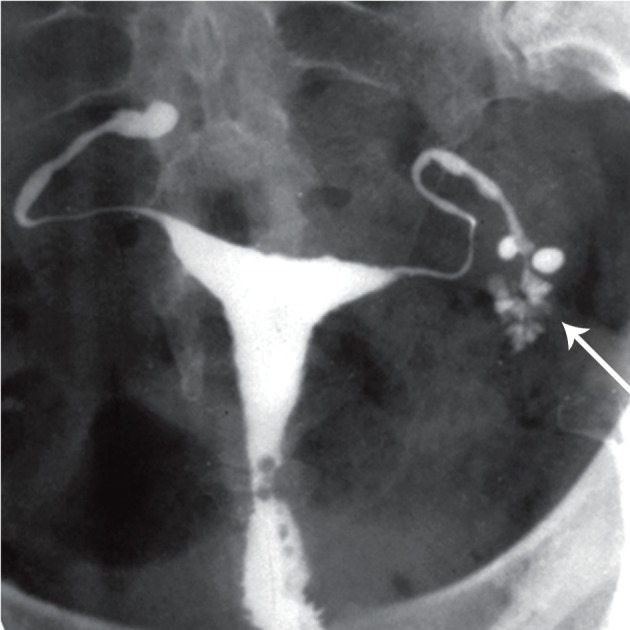
" Tufted tube" . Multiple small diverticular like appearance surrounding the ampulla produce d by caseous ulceration gives the tubal outline a Rosette-like appearance [arrow (32)].

**Fig 3:**
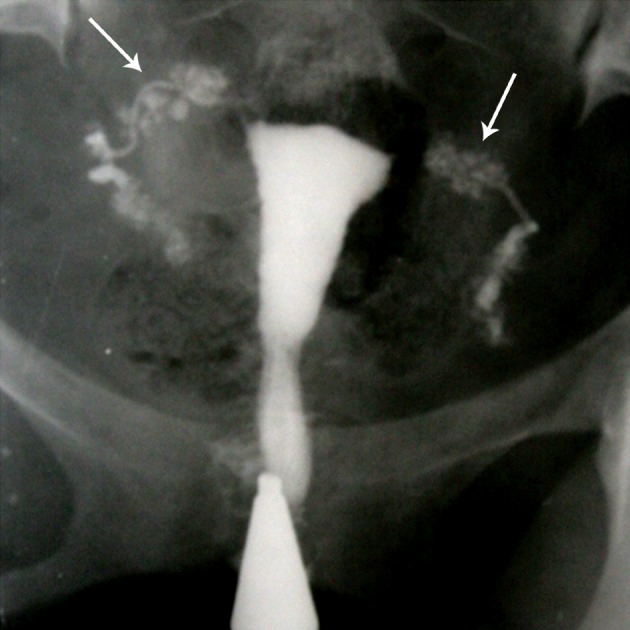
TB SIN-like. Penetration of contrast medium between the mucosal folds produces small diverticular-like outpouchings with a bizarre pattern. Entire of both tube involved (arrows). Moderate hydrosalpinx is seen in the right side (open arrow).

**Fig 4:**
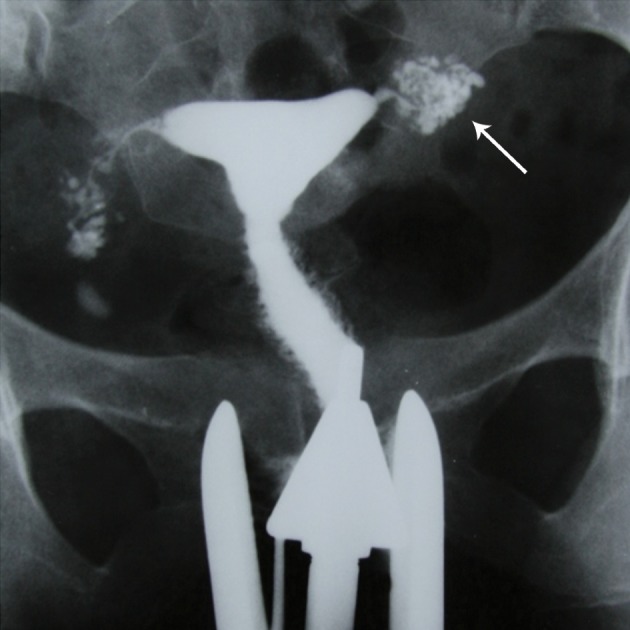
Distribution of contrast medium in a reticular pattern producing a " cotton-wool plug" appearance [arrow (32)].

**Fig 5:**
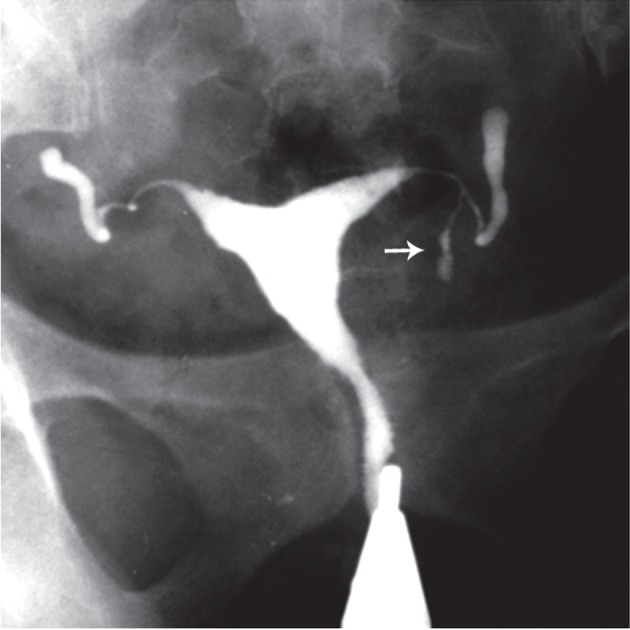
Terminal sacculation in both fallopian tubes. Moderate hydrosalpinx and blind ending sinus are seen in both tubes. Note the fistula in left tube [white arrow (32)].

Caseous ulceration of tubal mucosa creates an irregular, ragged or divertucular appearance on the contour of the tubal lumen on HSG. Divierticular cavities surrounding of the ampullar portion may give it a "tufted" like appearance (Fig 2). Isthmic diverticula may resemble salphingitis isthmica nodosa, "TB-SIN" like appearances can be differentiated from classic SIN (Fig 3). In "TB-SIN diverticular outpouching are larger, asymmetric, with a more bizarre pattern (in size and number) and are not usually restricted to the isthmic portion of the tube as compared with those of SIN (8, 16). The contrast shadow of tubal termination may not be clearly verified but dissemination of contrast in an irregular manner produce a cotton-wool plug appearance (Fig 4). When the tubal lumen is filled with putty-like caseous material the salpingographic outline is irregular with pockets or lacunae giving a sawtoothed appearance. A blind ending sinus tract or occasionally fistula to an adjacent bowel may form (Fig 5) (8, 32) .

### Tubal occlusion

Tubal occlusion in tuberculosis is considered the most common finding seen on HSG and occurs most commonly at the junction between the isthmus and ampulla. in the region of isthmus and ampulla. Although cornual occlusion following ampullar obstruction is the most common site of tubal occlusion caused with any factor, it is not so common in tuberculosis.

**Fig 6:**
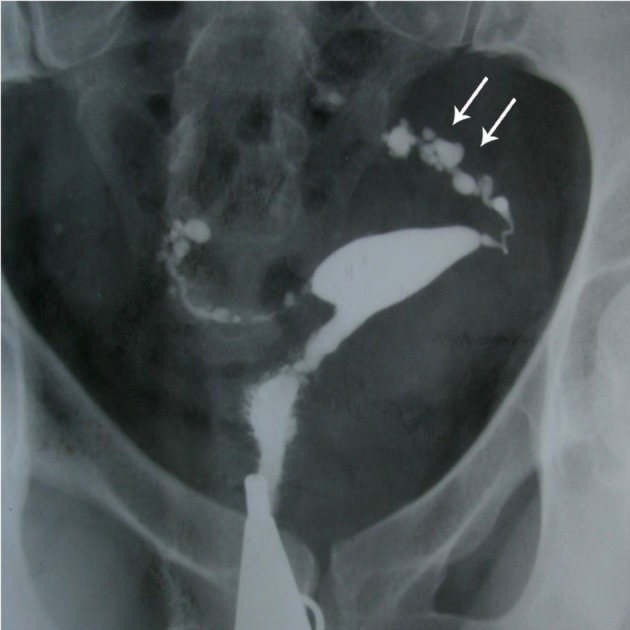
" Beaded tube" . Multiple constrictions along the fallopian tube giving rise to a " beaded" appearance [arrows (32)].

**Fig 7:**
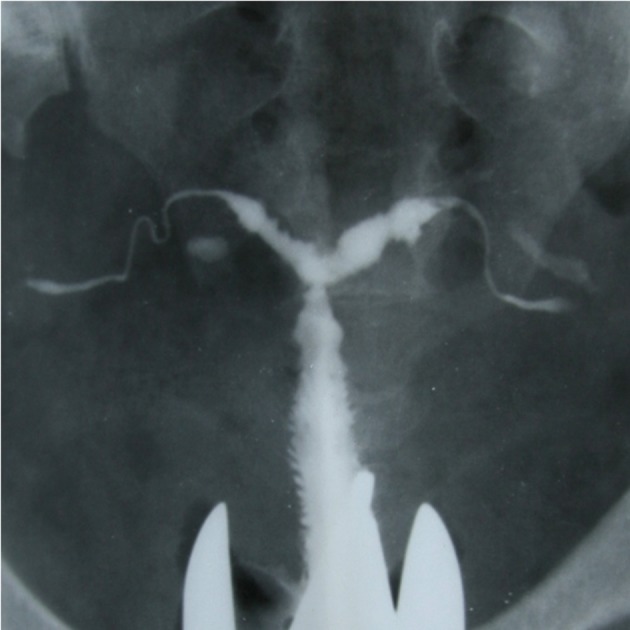
" Pipe stem" appearance in a women with primary infertility. Absence of normal tortuosity and a curved or straight pipe like appearance show fibrotic stage of tuberculous salpingitis. Irregular contour of the uterine cavity with diminished capacity in the fundual portion resembling a septate uterus.

Multiple constrictions along the course of fallopian tube may form due to scarring and present as "beaded" appearance (Fig 6). While tuberculosis gets better, the entire tube could be encased in a heavy connective tissue scar and the lumen then develops a "straight rigid pipe stem" appearance without normal tortuosity (Fig 7) (33).

### Tubal dilation

**Fig 8:**
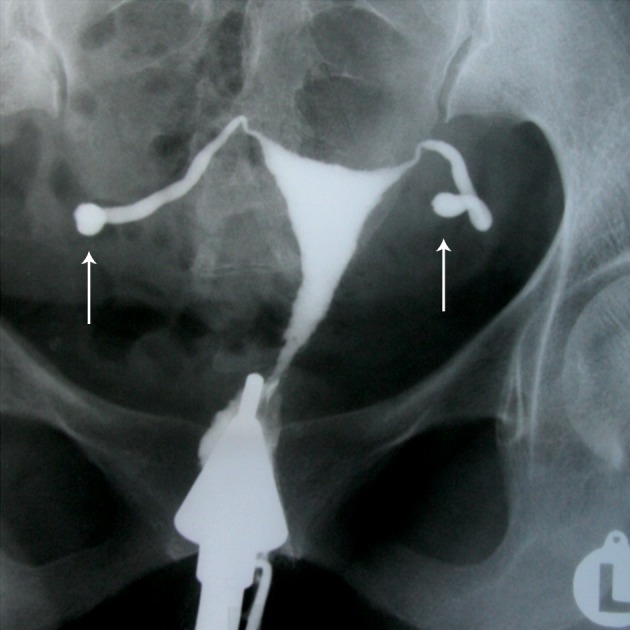
" Golf club" tube. Sacculation of both tubes in distal portion with an associated hydrosalpinx giving a Golf club-like appearance (arrows). Uterine cavity has normal size and shape.

Occlusion of the isthmus or fimbrial end of the tube filled with serous or clear fluid produce a retort-shaped dilation of the tube (large-sausageshaped) which initially is a pyosalpinx that change to hydrosalpinx. Hydrosalpinx is usually moderate or slight with a "golf club like appearance" to the ampulla (Fig 8) (16).

**Fig 9:**
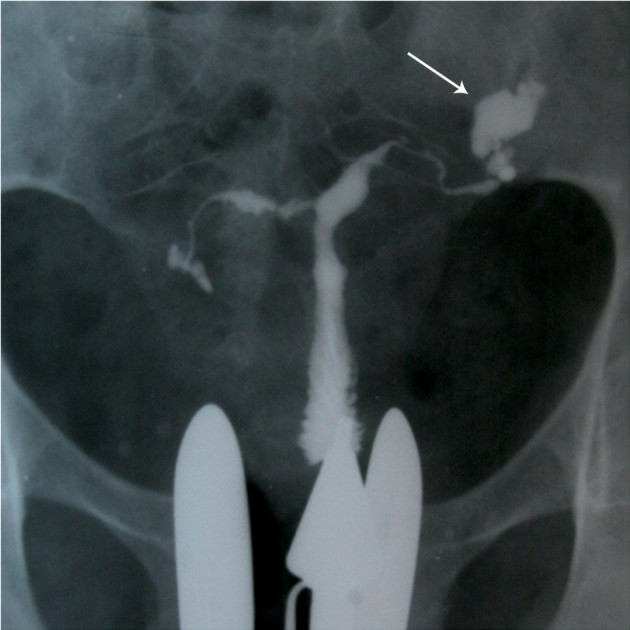
" Floral appearance" . Twisted hydrosalpinx resembles a floral appearance of left side tube (arrow).

**Fig 10:**
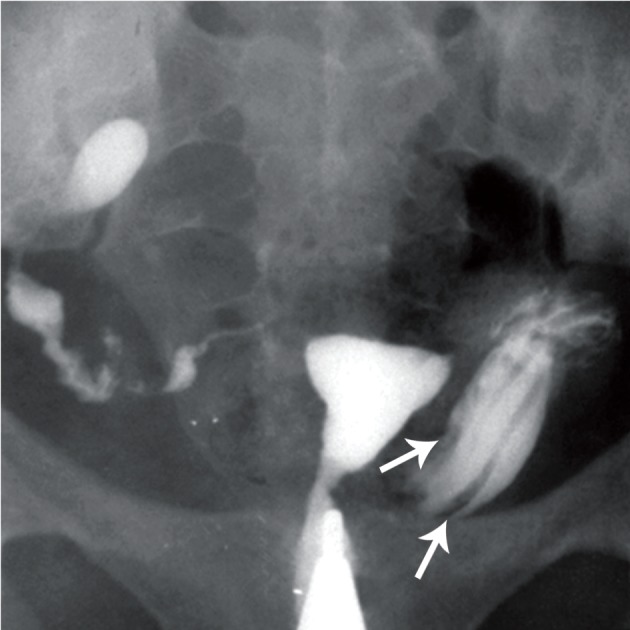
Thickened mucosal folds in the dilated tubes is a non-specific finding of tubal tuberculosis (32).

Twisting of the hydrosalpinx may result in a floral pattern- "the floral hydrosalpinx" (Fig 9). Thickening of mucosal folds in the dilated tubes, is another commonly feature which is seen in tuberculosis (Fig 10).

**Fig 11:**
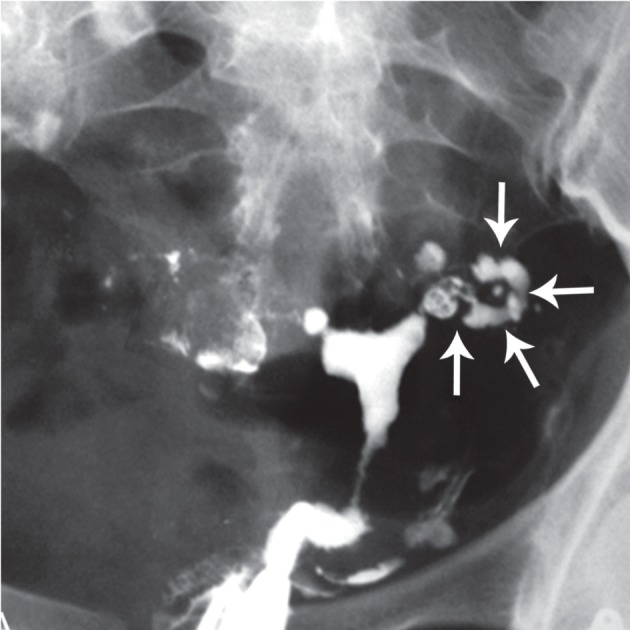
Cobblestone appearance (arrows). Intraluminal scarring of the tube gives rises a cobblestone like appearance which is an effective radiographic sign of intraluminal adhesions (32).

**Fig 12:**
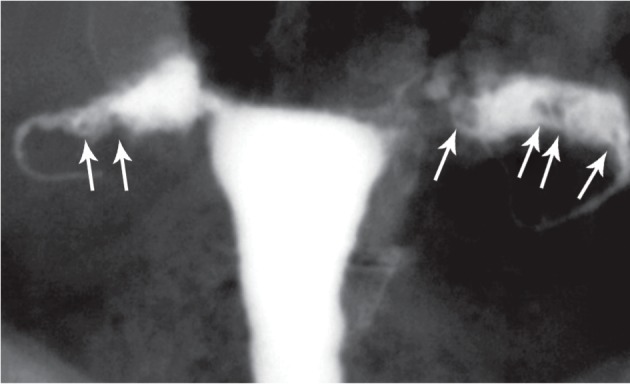
Multiple rounded filling defects following intraluminal granuloma formations within the hydrosalpinx, resembling a " leopard skin" appearance [arrows (32)].

Intraluminal scarring can give rise to a cobblestone pattern which is an effective radiographic sign of intraluminal adhesions in hydrosalpinges and associated with concern of infertility (Fig 11) (34). A similar appearance may be seen with multiple intraluminal granulomas formation. In this circumstance the ampulla is partially filled with dye and giving a spekled leopard skin appearance (Fig 12) (14).

### Peritubal adhesion

In chronic tuberculosis, following repeated episodes of acute exacerbation, a dense peritubal connective tissue scarring occurs in and around the tubes, leading to peritubal adhesions. The tubes become vertically or horizontally fixed, interfering with access of fallopian tubes to the ovary at ovulation and transport of the ovum.

**Fig 13:**
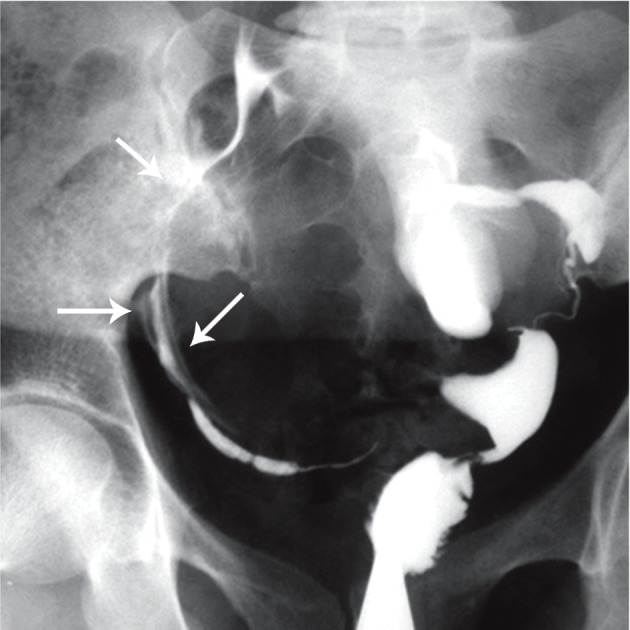
" Straight spill" pattern from a vertically fixed tube. Contrast spill is bounded by peritubal adhesions [arrows (32)].

**Fig 14:**
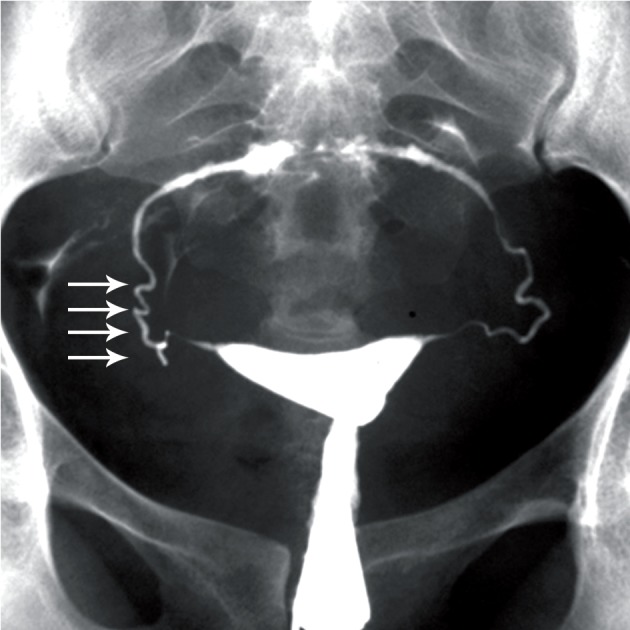
" Vertically fixed tubes secondary to dense peritubal adhesions. Dense connective tissue causes the lack of tubal mobility. The hyperconvulated is seen in right tube and manifests a " cork screw" like appearance [arrows (32)].

**Fig 15:**
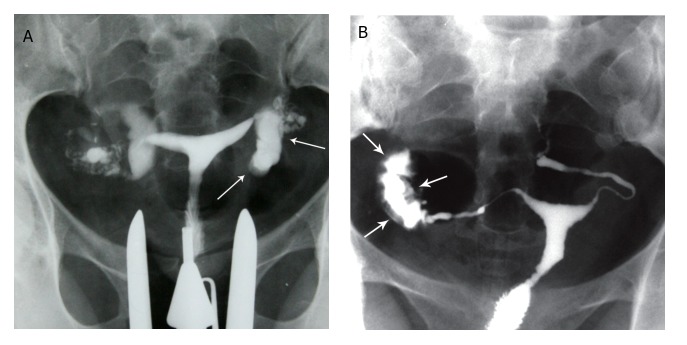
" A, B. Peritubal halo. Thickening of the tubal walls due to peritubal adhesions (arrows) represents a cloudy sign on hysterosalpingograms. This finding is a non-specific feature of tubal tuberculosis.

In this non-specific finding, the contrast spill from a vertically fixed tube appears to be bounded laterally by adhesions, which gives rise to straight spill appearance (Fig 13). The fallopian tube may also show a hyperconvoluted or corkscrew appearance (Fig 14). The presence of a convoluted or corkscrew fallopian tube, peritubal halo (cloudy appearance due to thickening of loculated tubal walls), tubal fixation and loculated spillage of contrast material is suggestive of peritubal adhesions (Fig 15) (35).

**Fig 16:**
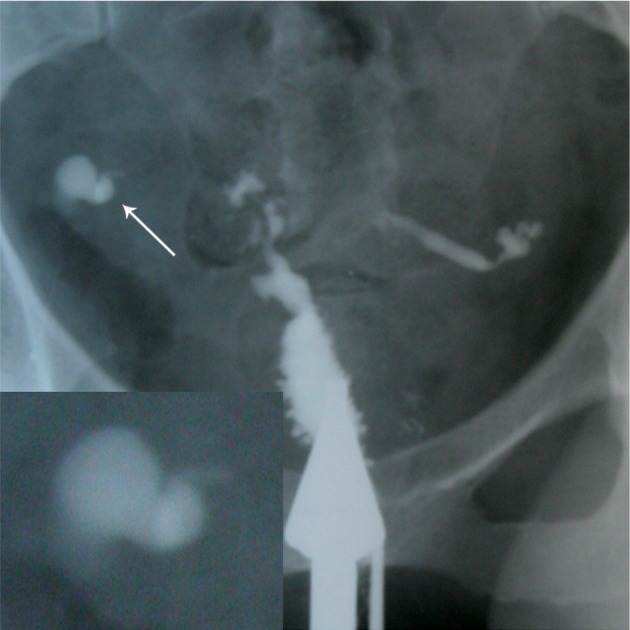
" Tobacco pouch" appearance, A. Terminal hydrosalpinx with the conical narrowing is seen in the right tube (arrow). Eversion of the fimbria secondary to adhesions, with a patent orifice produces the tobacco pouch appearance in the left terminal.

Dense adhesions may resemble lead to visualization of septations and bizarre "criss-cross spill" pattern. Sometimes, peritoneal granulomas formation produces small rounded filling defects seen additionally to these septations. Everted fimbria with a patent orifice imparting characteristic "tobacco pouch" appearance (Fig 16).

## Conclusion

HSG is considered as an important diagnostic tool in the investigation of internal architecture of female genital tract and helpful procedure in diagnosis of female genital tuberculosis. Tubal and uterine lesion scarring remained of genital TB, are presented as specific and non-specific radiographic features which should be differed from other pathological conditions. Since the incidence of genital tuberculosis has been increased during the past two decades, the clinicians increasingly faced with cases of genital TB and its consequences such as infertility, so reviewing of these features are considered in differential diagnosis of the causes of infertility and timing intervention and treatment.
